# Prevalence, incidence and residual risk of transfusion-transmitted HBV infection before and after the implementation of HBV-NAT in northern Brazil

**DOI:** 10.1371/journal.pone.0208414

**Published:** 2018-12-19

**Authors:** Angelita Silva de Miranda Corrêa, Letícia Martins Lamarão, Priscilla Cristina Moura Vieira, Renata Bezerra Hermes de Castro, Núbia Caroline Costa de Almeida, Jairo Augusto Américo de Castro, Maria Salete Maciel de Lima, Mauricio Koury Palmeira, Ana Luiza Langanke Pedroso Meireles, Rommel Rodríguez Burbano

**Affiliations:** 1 Laboratory of Nucleic Acid Test (NAT), Foundation Center for Hemotherapy and Hematology of Pará (HEMOPA), Belém, Pará, Brazil; 2 Laboratory of Human Cytogenetics, Institute of Biological Sciences, Federal University of Pará, Belém, Pará, Brazil; 3 Laboratory of Molecular Biology, Ophir Loyola Hospital, Belém, Pará, Brazil; Centers for Disease Control and Prevention, UNITED STATES

## Abstract

**Background:**

Nucleic acid testing (NAT) for virus detection during blood screening has helped to prevent transfusion-transmitted infections worldwide. In northern Brazil, NAT was implemented in 2012 for HIV and HCV and more recently, in January 2015, the screening for HBV was included and currently used concomitant with serological tests (HBsAg and anti-HBc). This study aims to evaluate the prevalence and the incidence of HBV infection among voluntary blood donors at ten regional blood centers of HEMOPA Foundation in Pará state and to compare the residual risk of transfusion-transmitted HBV infection before and after the Brazilian HBV-NAT implementation.

**Methods:**

The prevalence (restricted to first time donors- FT) and seroconversion rate (restricted to repeat donors- RP) of HBV were calculated based on rates of confirmed positive samples. Residual risk was based on the incidence and window period (WP) model described by Schreiber and coauthors. Logistic and Poisson regression were used in the statistical analysis by SPSS v20.0. A p value <0.05 was considered statistically significant.

**Results:**

HBV prevalence in the periods before and after the implementation of HBV-NAT were 247 and 251 per 100,000 donations, respectively. Seroconversion rates were 114 and 122 per 100,000 donations in the two periods, respectively. The residual risk (RR) for HBV decreased significantly in the posterior period to the HBV-NAT implementation, when compared to RR before implementation, with a reduction of 1:144,92 to 1:294,11 donations (p <0,001).

**Conclusions:**

The RR to HBV decreased after the implementation of HBV-NAT, increasing significantly the transfusional security in the North region of Brazil at HEMOPA Foundation.

## Introduction

Hepatitis B remains a major public health problem worldwide according to the World Health Organization approximately 240 million people have chronic hepatitis B and 470,000 deaths occur per year due to the consequences of hepatitis B such as cirrhosis and hepatocellular carcinoma [1].

Currently Brazil has approximately 207,000,000 inhabitants and the distribution of HBV is quite heterogeneous in the 8,516,000 km^2^ of country territorial extension. Aaccording to the Ministry of Health the incidence in the northern area is above the national average (11 vs. 6.5/100,000 inhabitants) [2]. However, southeast and south region concentrated the most cases of hepatitis B [3]. Although hepatitis B vaccine has been recommended nationwide since 2000 for newborns and individuals up to 49 years of age and vaccine coverage has had satisfactory results, there are still several barriers to reach the target population, such as regions with difficult access and lack of adherence to the complete vaccination schedule (three doses)[2,3].

Over the past decade, Brazilian hemotherapy has been characterized by the development of laboratorial technologies and by the adoption of standard protocols for donor screening. The set of quantitative and qualitative measures adopted improved the transfusion safety by minimizing transfusion risks, especially related to transfusion safety in transmissible infections. Nucleic acid testing (NAT) for virus detection during blood screening has helped to prevent transfusion-transmitted infection worldwide [4,5,6,7]. However, the risk of infection persists due to blood donations collected during the window period (WP) and the high prevalence of the disease in the source population, therefore some mathematical models are used to estimate the residual risk (RR) of transfusion-transmitted infections in several countries [8,9,10].

Brazilian technical regulations on hemotherapeutic procedures are determined by the Ministry of Health. The managing policies of blood products prohibit the monetary incentives to blood donors. The candidates go through an interview and medical evaluation where they answer a clinic and epidemiologic questionnaire. From the analysis of the candidate’s answers, the ones who demonstrate sexual behaviour with higher risks to sexual transmitted diseases will be excluded of the donation, following the American Association of Blood Banks (AABB) [11].

In Brazil, the importance of knowing and monitoring the risk of HBV transmission during a blood transfusion was due to the recent implementation of the national NAT platform for HIV, HCV and HBV detection, by Bio-Manguinhos (FIOCRUZ, Rio de Janeiro, Brazil) and Ministry of Health, in all Blood Centers of country. In the Center for Hemotherapy and Hematology of Pará (HEMOPA) Foundation–responsible for the most blood and hemocomponents transfused in the State of Pará (northern Brazil with approximately eight million inhabitants), it has been implemented in 2012 for HIV and HCV. Afterwards, in January 2015, the screening for HBV was included and now is currently used concomitant with serological test: HBV surface antigen (HBsAg) and antibodies to HBV core antigen (anti-HBc) [12].

Previous studies have reported a heterogeneous prevalence of the HBsAg marker between blood donors in different regions of Brazil, lower rates in the southern region, intermediate rates in the Northeast and Southeast, and higher rates in the Amazon region [10]. Prevalence and incidence data are important to have knowledge about the regional characteristics of HBV infection in blood donors and to assist in the development of public health policies. An accurate estimate of the risk of transfusion-transmitted HBV infection will help to monitor blood transfusion safety and will establish measures to minimize risk as well as assess the value of screening for HBV-NAT in Brazil. To our knowledge, this article is the first to evaluate the impact of HBV NAT implementation for blood screening in northern Brazil.The aim of this study was to determine the prevalence and incidence rates of HBV among voluntary blood donors of HEMOPA Foundation and to compare the residual risk of transfusion-transmitted HBV infection before and after the HBV-NAT implementation.

## Materials and methods

### Donors’ database

The present study was conducted at the main HEMOPA blood center (Belém city) and it was reviewed and approved by the Federal University of Pará Ethics and Human Research Committees number: 1.982.903 (CAAE: 65760817.0.0000.5634). This study included all the donations collected between 07/01/2013 and 06/30/2016 in the ten blood centers of the HEMOPA Foundation distributed in the north (Belém, Ananindeua and Castanhal cities), west (Santarém city), southeast (Marabá, Redenção and Tucuruí cities), southwest (Altamira city) and northeast (Abaetetuba and Capanema cities) of the state of Pará. The study period was divided in two to perform the analyses: before (07.01.2013–12.31.2014) and after (01.01.2015–06.30.2016) NAT implementation. The donor database includes age, sex, ethnicity, school level, results of the screening assays and number of seroconvertors for the anti-HBc and HBsAg markers. The donors were classified as first time (FT–donors without previous donation) and repeat donor (RP–donors with one or more donation per year). Donations were classified as replacement if blood donors were donating on behalf of a friend or relative or as community if their donation was not targeted to a specific person.

### HBV serological and molecular screening

Serological screening for HBsAg and anti-HBc markers were performed on all blood donations by the chemiluminescent assay (CMIA) using the Architect platform (Abbott Laboratories, Wiesbaden, Germany). Molecular screening for HBV was performed by NAT Kit manufactured by Bio-Manguinhos- detection limit of 50 IU/mL (FIOCRUZ, Brazil). The serological screening was applied in individual donations (ID) and the molecular screening was performed in a six-sample mini-pool (MP). NAT ID was applied to identify the positive samples in detected HBV MP. All Kits were applied according to the manufacturer's recommendations and all tests on blood samples were performed in the central laboratory at the main HEMOPA blood center, in Belém.

### Serological and molecular HBV confirmatory tests

The HBsAg marker (CMIA) was used to calculate prevalence rates in the period between 2013–2014. Only confirmed cases by the simultaneous presence of the total anti-HBc marker (CMIA) were included, and in the cases without this serological confirmation the presence of the viral antigen was confirmed by the PCR assay (Roche Molecular Diagnostics). In the period of 2015–2016, HBV-NAT was eligible for the prevalence calculation when concomitant with serological test (anti-HBc or HBsAg markers) or positive viral load test for those immunological window cases, performed by Bio-Manguinhos (FIOCRUZ, Rio de Janeiro, Brazil). The seroconversion rate was defined by the presence of anti-HBc in donors with negative prior donations. In a period before and after HBV-NAT implementation, the presence of anti-HBc was confirmed by the simultaneously presence of viral antigen (HBsAg in 2013–2014 and HBV-NAT in 2015–2016) or by the presence of anti-HBs by the enzyme immunoassay kit (EIA—Abbott Laboratories).

### Prevalence and seroconversion rate per 100,000

The HBV prevalence was restricted to FT donors. The calculation was based on positive donations for HBsAg between the years 2013 and 2014, and between the years 2015 and 2016 on donations detected by HBV-NAT. Positive donations were divided by the total number of donations before and after HBV-NAT implementation. The seroconversion rate was restricted to RP donors and it was defined as the number of confirmed seroconverters (anti-HBc total) divided by the number of donations before and after the NAT implementation. The HBV prevalence and seroconversion rate were calculated by the type of donor, type of donation, blood bank region and donor characteristics.

### Estimates of incidence person-year and residual risk

The study period was divided in before (18 months) and after (18 months) HBV-NAT implementation to perform the analyses. The incidence person-year (*py*) was calculated by dividing the number of RP donors who had the confirmed presence of HBV viral antigen (HBsAg or HBV-NAT) divided by the total number of *py* at risk. The number of *py* at risk was obtained by summing the days of the interdonation intervals between first and last donation for each RP donors. The time at risk for an uninfected donor is the time from first to last donation in the estimation interval and the time at risk for a seroconverting donor is the time from first donation in the estimation interval to half way between the last two donations. This calculation assumes that the risk of conversion is equally spread over the intervals; therefore their mid-point is the best unbiased estimate on this group.

The *py* incidence was adjusted considering that seroconversion to the hepatitis B surface antigen (HBsAg) will underestimate the incidence due to the transient nature of the antigenemia. In the period before HBV-NAT, the correction factor for HBsAg was estimated considering the positivity time of the 63-day HBsAg and the observed distribution of interdonation intervals among the study group, according to reports by Korelitz and co-authors [13]. In the period after HBV-NAT, the correction factor was estimated considering the mean of the seroconversion interval among the study group and the estimated period for detection of transient DNA of 94.5 days as suggested by Vermeulen and co-authors [5].

The estimates of the residual risk (RR) of transfusion-transmitted viral infection were based on the incidence *py* adjusted and WP estimates model described by Schreiber and coauthors [14]. The residual risk is obtained by multiplying the incidence *py* for the WP expressed as a fraction of the year, according to the following formula: RR = incidence *py* x (WP screening test/365 days). The serological WP was used to calculate the RR before the HBV-NAT implementation (38.3 days—obtained from previous report [15,16]) and the after the HBV-NAT implementation was used 12 days (according to the manufacturer's).

### Statistical analysis

Initially, the Pearson’s chi-square test was used to evaluate the association between the differences in the categorical variants of prevalence and seroconversion rates. Next, the adjusted multivariate logistic regression model was applied. The absence of multicollinearity between the variables was examined by means of the variance inflation factor (VIF), and the cutoff value adopted for the existence of multicollinearity was VIF≥ 4. Fisher’s exact tests and Pearson’s chi-square were used for comparison of Confirmed HBV Positive and Person-years before and after HBV-NAT implementation. Poisson regression was used to evaluate the *py* incidence and residual risk differences, and CIs assumed that incident cases are Poisson distributed and residual risk estimates were computed with a Taylor series approximation to the residual risk standard error estimates. Data were analyzed using a statistics package SPSS v20.0 (SPSS, Chicago, IL, USA). A *p* value of less than 0.05 was considered statistically significant.

## Results

During the period from July 1, 2013, to June 30, 2016, a total of 294,881 consecutive blood donations were collected at the ten Blood Centers of the HEMOPA Foundation. In the period before HBV-NAT implementation, 141,514 samples (which correspond to 47.9%) were screened for HBsAg and anti-HBc and after the implementation of HBV-NAT, 153,367 samples (52%) were tested concomitant by NAT and serological tests. A total of 208,780 (70.8%) of all donations came from repeat donors and was observed a predominance of community type donation (67.7%). The main donor group was composed by male (65.4%), aged between 16 and 39 years old (62.48%), high school level (59.24%) and mixed ethnicity (76.5%). Across all donations, 71.5% were from the North region of the state (Belém, Ananindeua and Castanhal cities) (Table 1).

**Table 1:** Donation characteristics.

Characteristics	Number	(%)
**All Period**	294,881	
Before HBV-NAT	141,514	47.99
After HBV-NAT	153,367	52.01
**Type of donnor**		
First Time	86,101	29.20
Repeat	208,780	70.80
**Sex**		
Male	192,918	65.42
Female	101,963	34.58
**Donation type**		
Replacement	95,125	32.26
Community	199,756	67.74
**Age group (years)**		
16–39	184,258	62.48
40–69	110,623	37.52
**Blood Center**		
North	210,952	71.53
West	19,608	6.64
Southeast	39,301	13.32
Southwest	9,037	3.06
Northeast	15,983	5.42
**School Level**		
Illiterate	291	0.1
Middleschool	48,566	16.47
High school	174,681	59.24
University	71,343	24.19
**Ethnicity**		
Black	10,720	3.63
Mixed	225,789	76.58
White	58,372	19.79

In the previous period to the implementation of HBV-NAT (2013–2014), 101 from the total of 108 FT donors detected by HBsAg were confirmed and included in this study. From these 101 included cases, 100 (99%) presented simultaneously anti-HBc and one presented the profile HBsAg+, anti-HBC- and PCR +. At the same period, 115 out of a total of 178 RP donors who seroconverted to anti-HBc were confirmed and included in this study. From these cases, 5 (4,3%) also presented the HBsAg and 100 presented the profile anti-HBc+, HBsAg- and confirmatory anti-HBs+.

In the period after the implantation of HBV-NAT (2015–2016), 114 FT donors were detected by the HBV-NAT. All of these cases were confirmed and included in this study. From this total, anti-HBc was also positive in 100% of the cases. HBsAg had coexisted in 108 of the cases (95%) and in 6 cases (5%) the occult hepatitis were confirmed (HBV-NAT +, anti-HBc + e HBsAg-). At the same time, 132 out of the total of 206 RP donors that seroconverted by anti-HBc were confirmed and included in the study. From these, 5 donors (3,8%) presented simultaneously HBsAg and HBV-NAT and the remaining 127 (96,2%) presented the profile anti-HBc+, HBsAg-, HBV-NAT+ and anti-HBs+ confirmatory. There were no occult hepatitis cases in RP donors in this period.

In Table 2 the prevalence per 100,000 donations in the period before and after HBV-NAT implementation were 247 and 251 respectively. In both periods, donations from male donors were more prevalent than donations from female, replacement donations showed higher prevalence than community donations, as the donor age group 40–69. In the period before HBV-NAT implementation, the Metropolitan region of the Pará State (North) presented low prevalence when compared to other regions. In the period after HBV-NAT implementation the Southwest blood center (Altamira city) presented the higher prevalence. There was no difference observed in the ethnicity group in both periods. After adjusted multivariate logistic regression analyses, the variables sex, donation type, age group, blood center and school level remained associated with HBV prevalence rate on blood donor in both periods.

**Table 2:** Prevalence rate of HBV infection per 100,000 donations in Pará state of Brazil, before and after HBV-NAT implementation.

	BEFORE HBV-NAT (2013–1014)						AFTER HBV-NAT (2015–1016)					
Variables	FT donations	N. HBsAg positive	Prevalence rate (95% CI)	p value	AOR (95% CI)	p value	FT donations	N. HBV-NAT Positive	Prevalence rate (95% CI)	p value	AOR (95% CI)	p value
**N. donations**	40,769	101	247 (203–300)				45,332	114	251 (209–301)			
**Sex**												
Male	22,837	69	302 (238–382)		1		24,805	78	314 (252–392)		1	
Female	17,932	32	178 (126–251)	0.01	0.58 (0.38–0.89)	<0.001	20,527	36	175 (126–241)	0.003	0.55 (0.37–0.82)	0.004
**Donation type**												
Replacement	12,496	41	328 (241–444)		1		15,386	54	350 (269–457)		1	
Community	28,273	60	212 (164–273)	0.025	0.64 (0.43–0.96)	0.04	29,946	60	200 (155–257)	<0.001	0.57 (0.39–0.82)	0.004
**Age group**												
16–39	30,316	63	207 (162–265)		1		33,700	70	207 (164–262)		1	
40–69	10,453	38	363 (264–498)	0.005	1.75 (1.17–2.62)	0.04	11,632	44	378 (281–507)	0.002	1.82 (1.25–2.66)	0.004
**Blood Center**												
North	29,122	37	127 (92–175)		1		32,471	50	153 (116–202)		1	
West	2,711	16	590 (363–956)		4.66 (2.59–8.40)		3,014	8	265 (134–522)		1.69 (0.80–3.56)	
Southeast	5,536	33	596 (424–835)	<0.001	4.71 (2.94–7.54)	0.002	5,931	35	590 (424–819)	<0.001	3.84 (2.49–5.93)	<0.001
Southwest	1,182	10	846 (460–1,550)		6.70(3.32–13.51)		1,455	20	1,374(891–2,113)		9.03 (5.36–15.21)	
Northeast	2,218	5	225 (96–526)		1.77 (0.69–4.52)		2,461	1	40 (7–229)		0.26 (0.03–1.90)	
**School level**												
Illiterate	42	1	2,380(421–12,321)		4.4 (0.59–32.77)		44	1	2,272(402–11,807)		3.44 (0.46–25.55)	
Middle school	7,815	43	550 (408–740)		1		6,709	45	670 (501–896)		1	
High school	23,813	51	214 (162–281)	<0.001	0.38 (0.26–0.58)	<0.001	27,313	52	190 (145–249)	<0.001	0.28 (0.18–0.42)	<0.001
University	9,099	6	65 (30–143)		0.12 (0.05–0.28)		11,266	15	133 (80–219)		0.19 (0.11–0.35)	
**Ethnicity**												
Black	1,532	8	522 (264–1027)		1		1,604	7	436 (211–898)		1	
Mixed	31,442	77	244 (196–305)	0.10	0.46 (0.22–0.97)	0.095	34,406	91	264 (215–324)	0.23	0.60 (0.28–1.30)	0.1
White	7,795	16	205 (126–333)		0.39 (0.16–0.91)		9,322	16	171 (105–278)		0.39 (0.16–0.95)	

Table 3 shows the results of seroconversion rate of HBV infection in the period before and after HBV-NAT implementation, which were estimated at 114 and 122 *per* 100,000 donations, respectively. The age group 16–39 showed a higher seroconversion rate in the period before HBV-NAT implementation, however, in the period after HBV-NAT implementation was observed the higher seroconversion rate in the age group 40–69. Southwest blood center (Altamira city) and the blood centers of Metropolitan region of the Pará State (North region) presented the higher seroconversion rate in the period before HBV-NAT implementation, however, in following period, the Southwest and the Southeast region presented higher rate. Only in the period after HBV-NAT implementation, donor attending high school level showed a higher seroconversion rate of HBV. After adjusted multivariate logistic regression analyses, the variables age group and blood center, remained associated with HBV seroconversion rate in blood donor in both periods.

**Table 3:** Seroconversion rate of HBV infection per 100,000 donations in Pará state of Brazil, before and after HBV-NAT implementation.

	BEFORE HBV-NAT (2013–1014)						AFTER HBV-NAT (2015–1016)					
Variables	RP donations	N. anti-HBc positive	Seroconversion rate (95% CI)	p value	AOR (95% CI)	p value	RP donations	N. Anti-HBc positive	Seroconversion rate (95% CI)	p value	AOR (95% CI)	p value
**N. donations**	100,745	115	114 (95–137)				108,035	132	122 (103–145)			
**Sex**												
Male	71,100	88	124 (100–152)		1		74,176	95	128 (105–157)		1	
Female	29,645	27	91 (63–132)	0.8	0.73 (0.48–1.13)	0.13	33,859	37	109 (79–151)	0.82	0.85 (0.58–1.25)	0.33
**Donation type**												
Replacement	30,576	38	124 (91–171)		1		36,667	45	122 (91–164)		1	
Community	70,169	77	110 (88–137)	0.97	0.88 (0.60–1.30)	0.69	71,368	87	121 (98–150)	0.7	0.99 (0.69–1.42)	0.92
**Age group**												
16–39	41,315	71	171 (136–216)		1		78,927	81	103 (83–127)		1	
40–69	59,430	44	74 (55–99)	<0.001	2.32 (1.60–3.38)	0.04	29,108	51	175 (133–230)	0.002	1.70 (1.20–2.42)	0.004
**Blood Center**												
North	71,974	95	131 (108–161)		1		77,385	69	89 (70–113)		1	
West	6,699	2	29 (8–109)		0.29 (0.07–1.17)		7,184	7	97 (47–201)		1.09 (0.50–2.37)	
Southeast	13,681	12	88 (50–153)	0.03	0.66 (0.36–1.21)	0.025	14,153	43	304 (225–409)	<0.001	3.41 (2.33–4.99)	<0.001
Southwest	2,921	4	137 (53–351)		1.03 (0.38–2.82)		3,479	11	316 (177–565)		3.55 (1.87–6.72)	
Northeast	5,470	2	37 (10–133)		0.27 (0.06–1.12)		5,834	2	34 (9.4–125)		0.38 (0.09–1.56)	
**School level**												
Illiterate	101	0	0		0		104	0	0		0	
Middle school	17,768	24	135 (91–201)		1		16,274	19	117 (75–182)		1	
High school	59,706	70	117 (93–148)	0.78	0.87 (0.55–1.38)	0.64	63,849	91	142 (116–175)	0.04	1.22 (0.74–2.00)	0.99
University	23,170	21	91 (59–138)		0.67 (0.37–1.20)		27,808	22	79 (52–120)		0.67 (0.37–1.25)	
**Ethnicity**												
Black	3,760	9	239 (126–454)		1		3,824	4	105 (41–269)		1	
Mixed	77,823	83	107 (86–132)	0.07	0.44 (0.22–0.88)	0.5	82,118	111	135 (112–163)	0.3	1.29 (0.48–3.5)	0.08
White	19,162	23	120 (79–180)		0.50 (0.23–1.08)		22,093	17	77 (48–123)		0.73 (0.24–2.19)	

A total of 5 RP donors were converted to HBsAg in the period before the implementation of HBV-NAT, all 5 cases also had simultaneously seroconversion to anti-HBc (Table 4). The person time during this period among all repeat donors was 103,376, presenting an HBV incidence rate of 4.83 (95% CI 2.06–11.32) *per* 100,000 *py*. The incidence *py* adjusted was 6.65 (95% CI 3.38–13.14). Using 38.3 days for the infectious WP, the RR of transfusion-transmitted HBV infection was estimated in 0.69 *per* 100,000 donations (95% CI 0.63–0.74) or 1:144,92 donations.

**Table 4:** *Py* incidence rates and residual risks for HBV before (2013–2014) and after (2015–2016) HBV-NAT implementation.

	Before HBV-NAT	After HBV-NAT	
	2013–2014	2015–2016	p value
**Window period (days)**	38.3*	**12**^**¥**^	
Confirmed HBV Positive	5	10	<0.001
Person-years	103,376	110,747	0.68
Incidence *py* adjusted (CI 95%)	6.65 (3.38–13.14)	10.43 (6.99–18.71)	0.027
Residual risk *per* 100,000 (CI 95%)	0.69 (0.63–0.74)	0.34 (0.28–0.39)	<0.001
Residual risk (1 in)	144,92	294,11	<0.001

* Window period obtained from previous report [15,16].

^¥^ Window period obtained according to the HBV-NAT kit manufacturer.

In the period after the implementation of HBV-NAT, 10 RP donors were detected with the presence of viral DNA, all of these cases were confirmed and included in this study. The number of confirmed cases of HBV was higher in this period (10 *vs*. 5 cases, p<0.001). Out of the total, 4 cases (40%) presented at the same time the marker HBsAg. Three cases (30%) presented two serological markers (HBsAg and anti-HBc) and the last three cases (30%) were considered WP cases. In this period the total person-years at risk was 110,747, producing an incidence rate of HBV of 9.02 (95% CI: 4.9–16.62) per 100,000 *py*. The adjusted incidence was 10.43 *per* 100,000 *py* (95% CI 6.99–18.71). This period also presented a higher incidence rate than the period before HBV-NAT implementation (10.43 *vs*. 6.65, p = 0.027). Estimated WP was reduced to 12 days using HBV-NAT, thus, the RR of transfusion-transmitted HBV infection was significantly reduced to 0.34 *per* 100,000 donations (95% CI 0.28–0.39) or 1:294,11 donations (p<0.001).

## Discussion

This study describes the prevalence and the incidence of HBV infection by serological and molecular markers and residual risk of transfusion-transmitted HBV among voluntary blood donations in northern Brazil.

Serological markers in the period of 2013–2014 and molecular markers in the period of 2015–2016 showed similar prevalence in both periods (247 and 251/100,000). The general prevalence of HBV among blood donors in this study was very high compared with other previous blood services in developed countries like Canadian Blood Services (97/100,000, 2001–2005) [16], and American Red Cross (70/100,000, 2001–2002) [17]. In Argentina the most current prevalence of HBV in voluntary blood donors was 198/100,000, lower rate than those found in our study [18].

In Brazil, the prevalence rates of HBV infection markers in blood donors shows significant numbers and varies according to the serological marker: 0.14%-0.68% for HBsAg and 2.05%-6.12% for anti-HBc [19,20]. A study conducted in three blood banks located in different cities of the country showed differences in the prevalence of HBV (HBsAg and anti-HBc reactivity) infection in FT donors. In Recife (northeast), a higher prevalence of infected donors was observed (419/100,000), followed by Belo Horizonte (270/100,000) and São Paulo (213/100,000), both cities in the southeast region [10]. Our study presented a lower prevalence in relation to the cities of Recife and Belo Horizonte and a higher prevalence than that found in the city of São Paulo. This data is important and it is a warning to local authorities to strengthen prevention, protection and treatment of Hepatitis B.

In Brazil, according to the Ministry of Health, the prevalence rate (in the presence of symptoms) in 2016 was estimated at 6.9 per 100,000 inhabitants in the general population. In the period from 1999 to 2016, 212,031 confirmed cases of HBV were reported, with the majority of these cases (HBsAg and anti-HBc reactivity) concentrated in the Southeast (35.5%) and South (31.4%) regions, followed by the North (14.3%), the Northeast (9.4%) and the Central West regions (9.3%) [2]. This same report showed in the last two years a growing notification of cases mainly in the north region [21]. However, the absence of notification cases of HBV infection, especially in less developed regions of the state, can become an important bias in northern region data.

In our study a higher prevalence of HBV infection was observed in male donors aged over 40 years and with low school level. This data is in agreement with other studies in blood donors worldwide [6,10,15,22]. The highest prevalence among male donors may be correlated with the behavioral aspects adopted by these donors. In Brazil, the low school level may be associated with poor socioeconomic conditions and more restricted access to health services. In older ages, this association may be a reflection of a cumulative effect of behavioral risks. The highest prevalence in replacement donations is in agreement with results found in other Brazilian studies [22].

Despite the increasing rate of anti-HBs reactive subjects due to the successful immunization program in Brazil the adoption of NAT for hepatitis B screening in Brazilian Blood Centers has helped to prevent transfusion-transmitted infection. In the state of São Paulo, at Sírio Libanês hospital, there were HBV-infected (through blood transfusion) patients, which happened through WP and HBV-NAT and it could have interdicted this infectious unit [23]. The first Brazilian case of the immunological window for HBV was identified through individual HBV-NAT screening that identified one first-time donor as having hepatitis B in WP, which was not released for transfusion [24].

It was observed in the HEMOPA Foundation that after the introduction of HBV-NAT in donor screening, the *py* incidence increased significantly, probably due to the greater sensitivity of HBV-NAT that detected 3 cases of HBV in donors in WP, strengthening the effectiveness of HBV-NAT in relation to the serological methods as well as the importance that this technique has added to the strategies to increase transfusion safety in our region.

The introduction of HBV-NAT test also allowed the detection of 6 occult hepatitis cases among the 45,332 FT donor samples in the period of 2015–2016. It is appropriated the data supply of prevalence of this clinic entity in blood donors in order to do an evaluation of the viral persistence state, nevertheless, occult hepatitis can be transmitted through transfusion mainly in those countries that did not adopt the anti-HBc test in serological triage.

In relation to the seroconversion rate of HBV in RP donors was observed that only during before HBV-NAT period (2013–2014) there was a bigger rate of donors who were among 16–29 (the lowest prevalence in FT donors to HBV), these data can reflect the necessity to invest in prevention and vaccine campaigns (as this age group shows more active sexual behavior), therefore being the most common infection through HBV.

The residual risk is a very important statistical tool applied in hemotherapy to analyze transfusion safety, it is expressed as the chance of contracting a certain infection through transfused blood. Therefore, the estimates of residual risk are critical to monitoring transfusion safety and assessing the potential effect of new screening tests in Blood Centers [13,25]. Our study estimated for the first time the residual risk of HBV transfusion-transmitted in Northern Brazil. The residual risk results showed a significant reduction, from 1: 144,92 to 1: 294,11, due to the fact that the WP of the HBV-NAT is shorter than the WP of serological tests. As a result, this data showed that there was an improvement in transfusion safety in our region.

In Brazil, before HBV-NAT implementation, Kupek and coauthors estimated in the south of the country the RR for HBV by different methods (stand-alone HBsAg, HBsAg yield method, and recent anti-HBc seroconversions). The RR estimated ranged from 1:30,821 to 1:62,482 [26].

The residual risk for HBV transfusion-transmitted found in our study is very high when compared to developed countries [15]. The countries of the Americas and European are considered by the WHO to be of low prevalence for HBV [1]. Blood services estimated the residual risk before HBV-NAT ranging from 1: 62,500 to 1: 640,000 and after HBV-NAT ranging from 1: 115,000 to 1: 8,000,000 [15,27,28,29,30,31]. Considered by WHO as regions of medium and high prevalence of HBV infection, respectively, the Republic of Korea and the African continent described the RR before HBV-NAT between 1: 121 and 1: 1,000 and after HBV-NAT, Republic of Korea and the South Africa, between 1: 39,956 and 1: 43,666 [32,33,34,35,36,37].

Blood transfusion is considered a potential risk factor for transmission of viral infections [38], therefore the first step for proper recruitment for blood safety is to reduce the risk of transfusion-transmitted infections, avoiding collecting blood from donors with higher risk and preventing the waste of resources. Secondly, the effectiveness of the pre-donations interview to eliminate candidate donors who engage in risky sexual behavior. Therefore, an approach during clinical screening that could, above all, during the care, raise awareness, clarify and sensitize the donor about the importance of the act, can reduce these risks [39].

We recognized some limitations in our study. HBV seroconversion rate may have been underestimated since only donations with positive confirmatory tests were considered in the study. This criterion adopted in our study was able to avoid false-positive results. According to the literature, the genotype can influence WP [7]. Our study did not investigate which genotypes were circulating during this period in our region, however, a recent study revealed that the most frequent genotypes in Brazil are A (58.7%), D (23.4%) and F (11.3%) [40].

## Conclusion

The safety of blood transfusion in northern Brazil has increased significantly with the deployment of HBV-NAT, contributing to greater safety of blood products produced at the HEMOPA Foundation. However, recruitment strategies and donor selection remain important tools that need to be improved to ensure, along with screening tests, a better transfusion safety. In addition, the Brazilian NAT platform aims to increase the sensitivity of the kit as well as the inclusion of new targets.
